# Vaccines in Development against West Nile Virus

**DOI:** 10.3390/v5102384

**Published:** 2013-09-30

**Authors:** Samantha Brandler, Frederic Tangy

**Affiliations:** Unité de Génomique Virale et Vaccination, INSTITUT PASTEUR, 28 rue du Dr Roux, Paris 75015, France; E-Mail: ftangy@pasteur.fr

**Keywords:** West Nile vaccine, West Nile virus, measles vaccine, recombinant live attenuated vaccine, flavivirus

## Abstract

West Nile encephalitis emerged in 1999 in the United States, then rapidly spread through the North American continent causing severe disease in human and horses. Since then, outbreaks appeared in Europe, and in 2012, the United States experienced a new severe outbreak reporting a total of 5,387 cases of West Nile virus (WNV) disease in humans, including 243 deaths. So far, no human vaccine is available to control new WNV outbreaks and to avoid worldwide spreading. In this review, we discuss the state-of-the-art of West Nile vaccine development and the potential of a novel safe and effective approach based on recombinant live attenuated measles virus (MV) vaccine. MV vaccine is a live attenuated negative-stranded RNA virus proven as one of the safest, most stable and effective human vaccines. We previously described a vector derived from the Schwarz MV vaccine strain that stably expresses antigens from emerging arboviruses, such as dengue, West Nile or chikungunya viruses, and is strongly immunogenic in animal models, even in the presence of MV pre-existing immunity. A single administration of a recombinant MV vaccine expressing the secreted form of WNV envelope glycoprotein elicited protective immunity in mice and non-human primates as early as two weeks after immunization, indicating its potential as a human vaccine.

## 1. Introduction: West Nile Virus Dissemination

West Nile virus (WNV), originally identified in 1937 from a febrile woman in the West Nile district of Uganda [1], is a flavivirus belonging to the Japanese encephalitis serocomplex in the *Flaviviridae* family, together with St Louis encephalitis (SLE), Murray Valley encephalitis (MVE), Japanese encephalitis (JE) and Kunjin (KUN) viruses. Since its first description, sporadic outbreaks have been reported in Africa, Asia, in Europe and the Mediterranean Basin. Usually asymptomatic in humans, with some case, fatalities were caused by encephalitis reported in Israel in the 1950s. From 1996, severe outbreaks with a high incidence of neurological disease and death were reported in Morocco, Tunisia, Italy, Russia, Israel and France [2]. In 1998, a virulent WN strain from lineage 1 was isolated in Israel from a stork with clinical symptoms of encephalitis and paralysis [3], and in 1999, a closely related lineage 1 strain of WNV strain was introduced for the first time in the New York City area (NY99 strain). Since then, WNV distribution extended, and by 2003, WNV was endemic in all of North America [4]. Clinical manifestations range from uncomplicated fever to fatal meningo-encephalitis. Although 80% of infections are asymptomatic, patients may develop clinical signs, including fever, headache, fatigue, aches and, occasionally, skin rash. According to the Centers for Disease Control and Prevention (CDC), less than 1% of infected people develop severe neuroinvasive disease, such as encephalitis, meningitis or poliomyelitis-like syndrome, with about 10% of cases being fatal [5]. The year 2003 was recorded as the worst WNV outbreak in the USA, with more than 9,000 cases and 265 fatalities. During the last 15 years, increasing numbers of WNV outbreaks have been associated with the appearance of more neuroinvasive strains and higher human fatalities. Nevertheless, until last year, most cases and fatalities reported were sporadic. In 2012, the US experienced the second worst WNV outbreak, with a total of 5,387 cases of WNV disease reported, with a higher proportion of neuroinvasive cases, including 243 deaths, one third of all cases being reported in Texas [5]. The same year, WNV cases were reported worldwide, with more than 50 cases reported in Italy, Israel, Russia, Serbia and Tunisia and Greece, with 167 cases [6]. 

Birds, particularly corvids, are the main reservoir for WNV, and migratory birds play an important role in virus spreading. Ornithophilic *Culex* ssp. mosquitoes are responsible for the accidental infection of humans and horses, which are considered as dead-end hosts [4]. The peak season generally occurs during late summer, corresponding to the return of migratory birds. WNV transmission has been occasionally documented after blood transfusion or organ transplantation [7], via breastfeeding [8] or intra-uterine exposure [9]. Since 2003, blood banks have screened for WNV presence in donors’ blood, enabling an accurate follow-up of WNV activity in communities. 

WNV is an enveloped spherical virus of 50 nm diameter that encloses a positive sense, single-stranded genomic RNA of approximately 11,000 nucleotides-long. Phylogenetic analyses describe five distinct WNV lineages [10]. Strains of lineage 1, responsible for fatal neuroinvasive disease in humans and animals, include the NY-99 strain introduced in North America [11] and the closely-related Israel 1998 strain [12]. Lineage 1 strains are also found in Africa, Asia, the Middle East, Europe and Australia (Kunjin subtype). Lineage 2, considered as a zoonosis in sub-Saharan Africa and Madagascar, has recently been introduced in Greece, Hungary and Italy and is associated with severe neuroinvasive infections in birds, horses and humans [13,14]. Lineage 3 has been isolated once in Czech Republic, lineage 4 strains, in Russia [15], and linage 5 strains have only been described so far in India [16]. 

The most recent WNV outbreaks are characterized by an increased proportion of neurological disease in both humans and horses [17]. Although veterinary vaccines are commercialized to protect horses [18], no licensed vaccine is available to prevent WNV infection in humans. The main reason for that is that pharmaceutical companies claim that the market is limited for a human vaccine, and the seasonal and unpredictable nature of the infection makes the set-up of clinical trials difficult.

## 2. West Nile Virus Vaccine Requirements

A WNV vaccine needs to protect against all WNV genotypes, particularly after the emergence of the neuroinvasive strains of lineage 2 in Europe. Recent evaluation of the Recombiteck^®^ canarypox vaccine expressing WNV pre-membrane and envelope proteins (PrM/E) derived from lineage 1 indicates that cross protection can be achieved against pathogenic strains of lineage 2 [19], and on the reverse, that vaccines derived from lineage 2 strains protect against both homologous and heterologous WNV strains [20]. The most susceptible populations to WNV disease are the elderly and immuno-compromised individuals. Therefore, WNV vaccines need to be developed towards these target populations and will require safety and efficacy trials in these particular groups. The correlate of protection for flavivirus vaccines is the induction of neutralizing antibodies, as demonstrated by the vaccines against Yellow Fever, Japanese encephalitis and Tick-borne encephalitis viruses [21]. The viral envelope (E) protein is the major target of virus neutralizing antibodies and is, therefore, the choice antigen to elicit protective immunity against flavivirus [21]. The crystal structure of the E protein has revealed that it is organized in three distinct ectodomains: domain I (EDI), domain II (EDII) and domain III (EDIII) [22,23], as previously described for dengue virus [24,25,26] and tick-borne encephalitis virus [27]. The central EDII contains immunodominant epitopes close to the fusion loop [28]. These epitopes elicit cross-reactive sub-neutralizing antibodies that can favor the risk of antibody-dependent enhancement (ADE) in Fc-receptor-bearing cells after flavivirus infection, potentially leading to severe disease [29]. On the opposite side, the C-terminal immunoglobulin-like EDIII, a 100 amino-acid sequence stabilized by a disulfide bridge, is involved in receptor binding and contains critical neutralizing epitopes able to block attachment and post-entry viral steps [30,31]. 

Recent studies aiming to characterize the humoral immune response triggered by flavivirus infection and epitope mapping of human and non-human primate monoclonal antibodies indicate that other non-EDIII neutralizing epitopes are located in the inter-domain regions of the flavivirus envelope. Complex quaternary structured epitopes exposed on the flavivirus envelope have also been described [32]. These epitopes, present in the intact virions, but not in the soluble recombinant E protein, are composed of amino acid residues that interact with both monomers in the E dimer at the hinge region between EDI and EDII [33,34]. Antibodies directed at these epitopes neutralize the virus in a post-attachment step, likely by inhibiting trimer formation, which is required for the acidic pH-induced fusion with the endosomes [35]. Besides, strongly neutralizing epitopes buried in the E structure seem to become accessible in a temperature-dependent manner, indicating that the structure is less rigid than previously thought [36].

## 3. West Nile Virus Veterinary Vaccines

Several licensed veterinary vaccines are currently available (Table 1). Two are inactivated whole WNV vaccines: the West Nile Innovator, a whole formalin inactivated WNV-based vaccine, developed by Fort Dodge Animal Health, and commercialized by Pfizer [37], and Vetera vaccine (Boehringer Ingelheim). A West Nile-Innovator DNA vaccine (Fort Dodge Animal Health, commercialized by Pfizer,) approved in 2005 has now been discontinued. A recombinant canarypox-vectored vaccine expressing the pre-membrane (PrM) and the E protein of the NY99 strain (Recombiteck Equine West Nile Virus Vaccine, Merial-Sanofi Aventis) is also commercialized [38,39]. A chimeric vaccine based on the backbone of Yellow-Fever 17D human vaccine expressing the PrM and E genes of the NY99 strain, ChimeriVax-WN01, was licensed in 2005 by the United States Department of Agriculture,USDA (Prevenile, Intervet), but in 2010, several marketed serials were recalled after an increased incidence in the number of adverse events following vaccination, including severe cases of anaphylactic reactions, respiratory disease and even death, were reported. All the vaccines currently commercialized require two to three injections and an annual immunization boost.

**Table 1:** West Nile virus (WNV) veterinary vaccines. PrM, pre-membrane.

Vaccine	Antigen
West Nile Innovator–Pfizer	Whole inactivated WNV [37]
Vetera vaccine–Boehringer Ingelheim	Whole inactivated WNV
West Nile-Innovator DNA Pfizer (discontinued)	Plasmid DNA PrM/E
Recombiteck–Merial	Canarypox expressing PrM/E [38,39]
Prevenile–Intervet (recalled)	YF17D backbone expressing WNV PrM/E

## 4. West Nile Virus Human Vaccines

Although there is still no human WNV vaccine approved today, several vaccine candidates are in development (Table 2). However, despite the demonstration of strong efficacy in animal models for most of them, only a few candidates have been tested in humans so far and only in early phase I trials, leaving no hope of marketing before 5–6 years, in the best case.

### 4.1. Vaccines under Clinical Trials

The ChimeriVax-WNV based on Yellow Fever 17D (YF-17D) live attenuated human vaccine, from which the PrM and E genes of Yellow fever virus (YFV) have been deleted and replaced by those of the WNV NY99 strain, was commercialized as a veterinary vaccine (ChimeriVax-WN01). To further attenuate this vaccine for human use, three mutations in the E protein (E107 Leu > Phe, E316 Ala > Val, E440 Lys > Arg) responsible for the attenuation of the SA14-14-2 Japanese encephalitis vaccine in equivalent positions were introduced, leading to the ChimeriVax-WN02 vaccine. Phase I clinical trials in a population aged 18–40 years old demonstrated the safety of this vaccine. All vaccinated individual developed neutralizing antibodies after a single immunization with 10^3^ or 10^5^ plaque forming units, and most of them mounted a specific memory T-cells response directed against an immunodominant epitope of the WNV E protein [40,41]. For phase II trial, the vaccine has been further plaque-purified to select a small plaque (SP) variant of ChimeriVax-WN02 encoding a mutation in the membrane protein (Leu > Pro) with a limited viremia profile. The safety and immunogenicity of this vaccine was recently demonstrated in adults aged 41–64 years and >65 years, with a small increase in viremia in the latter group [42,43]. Another Chimeric vaccine based on dengue 4 virus backbone (WN/DEN4-3’delta30) has been shown to be strongly immunogenic in mice, geese and monkeys [44] and is currently being tested in clinical trials [45]. While the ChimeriVax-WN02 has been shown to be unable to replicate in mosquitoes, the Chimeric WN/DEN4-3’delta30 virus can be transmitted by one of both WNV and dengue virus (DENV) vectors, *Aedes albopictus* [46], raising safety concerns. 

A DNA vaccine encoding the pre-membrane and E glycoproteins of the NY99 strain of WNV under the transcriptional control of the CMV/R promoter (cytomegalovirus enhancer/promoter with the human T-cell leukemia virus type 1 R region) was evaluated in phase I on 30 healthy adults, half aged 18–50 years and half aged 51–65 years. The three-dose vaccine regimen given at 21-day intervals was safe and induced CD4/CD8 T-cells and neutralizing antibodies in the majority of subjects [47,48]. 

Another vaccine candidate (WN-80E) based on the expression in *Drosophila melanogaster* of the soluble form of the E protein lacking the transmembrane domain was also tested in a phase I trial. This recombinant protein was previously shown to induce protective neutralizing antibodies in several animal models, including non-human primates [49,50,51,52]. In phase I clinical trial, three injections of low (5 µg), medium (15 µg) or high doses (50 µg) of the protein formulated with 3.5 mg of Alhydrogel’85 were tested [53]. The safety study indicated that the vaccine was well tolerated regardless of the dose. All the volunteers developed neutralizing antibodies two weeks after the third dose. Several subjects that received medium (15 µg) or high (50 µg) doses had neutralizing antibodies after the second dose [54]. 

Until now, the few vaccines that have entered in clinical trials either require three doses to provide a strong protective immunity or are based on strategies that may face difficulties in terms of safety before licensing. There is, therefore, a need to pursue the development of safe and strongly immunogenic strategies that may only require one or two immunizations to confer rapid protection in the case of an epidemic.

**Table 2:** West Nile virus vaccines in clinical trials.

Vaccine name	Antigen	Clinical trial
ChimeriVax-WN02	Chimeric YF17D backbone expressing WNV PrM/E	Phase II [40,41,42,43].
Chimeric WN/DEN4-3’delta30	Chimeric DV4 backbone expressing WNV PrM/E	Phase I [45].
Clinical trial VRC_303_	Plasmid DNA expressing PrM/E	Phase I [47,48].
WN-80E	Soluble E lacking the trans membrane domain	Phase I [49,50,51,52].

### 4.2. Vaccines in Preclinical Development (Table 3)

#### 4.2.1. Subunit Vaccines

A recombinant subunit vaccine candidate based on the soluble truncated form of WNV E protein produced in *E. coli* [55] induced neutralizing antibodies in mice and horses and protected mice from WNV challenge when combined with aluminum hydroxide [56]. Another recombinant truncated WNV-E protein produced in a baculovirus expression system also induced WNV neutralizing antibodies that protected mice and hamsters from WNV challenge [57]. Recombinant baculovirus expressing the premembrane and E proteins of WNV induced also a strong immune response in mice after two doses administered intramuscularly [58]. The truncated E protein delivered with poly-DL-lactide-coglycolide (PLGA), an inflammasome-activating nanoparticle covered with LPS [59] or in combination with CpG oligodeoxynucleotides to target Toll-like receptor TLR9 [60] raised a robust Th1-biased humoral response, as compared to Th2 responses induced when aluminum hydroxide was used as the adjuvant, and better protection from WNV encephalitis in mice [60]. The EDIII of flaviviruses, which contains the receptor binding epitope and is a major target of virus-neutralizing antibodies, has been identified as a promising candidate antigen for the development of recombinant subunit vaccines [30,31]. Several studies indicate that recombinant EDIII protein can induce immune responses that protect mice from WNV infection when adjuvanted with CpG oligodeoxynucleotides [19,61] or in fusion with bacterial flagellin, a toll-like receptor-5 ligand [20]. A continuous B-cell epitope derived from EDIII (aa 355–369) of WNV envelope protein (named Ep15) has been fused to HSP60 p458 peptide as the carrier. Mice immunized with this chimeric peptide were resistant to lethal challenges with three different WNV strains [62]. However, this vaccine candidate, limited to a single epitope, might easily lead to the selection of escape mutants if used as a single antigen. A conjugate vaccine based on recombinant EDIII covalently linked to virus-like particles (VLP) derived from bacteriophage AP205 was also recently proposed. A single injection of this vaccine with alum was sufficient to induce virus-neutralizing antibodies and provide significant protection from WNV challenge. Three injections of the vaccine completely protected mice when given either in the presence or absence of alum [63]. VLPs of WNV generated in insect cells by the expression of pre-membrane and envelope proteins (PrM/E) or capsid, pre-membrane and envelope proteins (C-prM/E) have also been shown to induce protective immunity in mice [64]. The last strategy was recently proposed based on retrovirus VLPs consisting in a retroviral Gag polyprotein forming the VLP scaffold and the CD16 ectodomain fused to the gamma chain of the high affinity IgE receptor as the pseudotyping platform. Substitution of the CD16 ectodomain by the EDIII of WNV or DENV E glycoproteins allowed the exposure of EDIII at the surface of the VLPs and the induction of a neutralizing antibody response in mice [65]. 

#### 4.2.2. DNA Vaccines

The DNA vaccine strategy is particularly suitable for WNV immunization. Indeed, the first DNA vaccine commercialized in 2005 was the veterinary West Nile Innovator DNA vaccine that expresses the WNV PrM-E protein able to assemble into VLPs *in vivo*. A human DNA vaccine derived from this platform was proven immunogenic in phase I clinical trials [40,41]. After an initial study demonstrating that intramuscular administration of a DNA expressing WNV PrM and E was able to protect mice and horses from lethal challenge [66], several other WNV DNA vaccine strategies have been proposed. They include the co-administration of a DNA vaccine expressing prM-E with an inactivated vaccine [67], a DNA vaccine expressing a consensus WNV EDIII co-delivered with an optimized IL15 adjuvant plasmid to enhance T-cell memory [68] or the co-expression in DNA of WNV antigens with lysosome-associated membrane protein (LAMP) to target the MHC-II compartment [69]. DNA vaccine candidates expressing a WNV complete genome mutant lacking the C protein to disable the formation of infectious particles [70] or expressing the full genome of the naturally attenuated Kunjin virus have also been described [71]. The same group developed a novel DNA vaccine platform based on capsid-deleted Kunjin DNA vaccine and the co-expression of the capsid protein from a separate promoter, enabling *in vivo* expression of single round infectious particles (SRIPs), similar to the RepliVax technology described below [72]. This strategy improves the immunogenicity of the vaccine candidate in mice and horses and the protective efficacy in mice compared with an otherwise identical construct that does not encode capsid [72]. Even though DNA vaccines are convenient for manufacturing purposes, their efficacy in humans remains a key issue. Strategies, such as the split-genome vaccine [72] that closely mimics live viral infection without producing infectious virus, may be particularly useful to trigger an efficient immune response by DNA-based vaccines against flaviviruses.

#### 4.2.3. Non-Replicating Single-Cycle Vaccines

Non-replicating single-cycle vaccines, such as RepliVAX WN, are based on the expression of WNV complete genome deleted from the capsid protein gene that can therefore initiate the viral infection cycle and produce PrM/E-containing sub-viral particles (SVPs) in packaging cells expressing the C protein in *trans* without production of infectious progeny [73,74,75,76,77,78]. RepliVAX WN has been modified to avoid the risk of recombination with wild-type WNV [75]. A single immunization with RepliVAX WNV in mice and hamsters provided protection from lethal WNV challenge [77,78], and RepliVAX WN elicited a protective immune response in non-human primates [74]. Another replication-incompetent vaccine based on an adenovirus vector expressing the C, PrM, E and NS1 genes from WNV has been shown capable of inducing both neutralizing and cellular immune responses against the E and the conserved C and NS1 antigens [79].

#### 4.2.4. Inactivated Virus Vaccines

The WNV-Innovator veterinary vaccine administered to horses is prepared by chemical inactivation of the highly virulent WNV-NY99 strain. In Japan, a formalin-inactivated WNV vaccine (WN-VAX) also derived from the WNV-NY99 strain grown in Vero cells, induced complete protection in mice lethally challenged with the WNV NY99 strain [80]. Another formaldehyde-inactivated vaccine based on the ISR98 WNV strain propagated on retina-derived PER.C6 cells (a cell line derived from human embryonic retinal cells transformed with the Adenovirus Type 5 (Ad5) E1A and E1B genes ) was shown to be protective in geese, with protection levels correlating with neutralizing antibody titers [81]. Even though this strategy elicits protective immune responses, its development may face important manufacturing costs, due to the need of biosafety level 3 facilities required for virus production, added to the requirement of complete inactivation [37]. To avoid these constraints, it was proposed to use the Kunjin virus (KUN), a naturally attenuated strain of WNV isolated in the 1960s in Northern Queensland, Australia, which shares 98% amino-acid of sequences with the virulent NY99 strains, including all the neutralizing epitopes [82,83]. No human fatal case has been documented after WN-KUN infection, albeit 2.5% of northern Queensland’s population is seropositive for this virus. Even though rare cases of encephalitis have been described after WN-KUN infection, WN-KUNV gives far less severe infections than the NY99 strain in both humans and animals [84]. A recently developed hydrogen peroxide (H_2_O_2_)-inactivated virus based on the WN-KUN strain has been reported to generate a robust adaptive B- and T-cell immune responses in both young and aged vaccinated mice that were subsequently protected against lethal intracranial challenge with a heterologous virulent NY-99 WNV strain [85]. Another strategy has been proposed that combines six contiguous cDNA fragments encoding the genome of the NY99 strain to construct a bipartite infectious clone. This system is designed to reduce the emergence of sequence variants during the production process. The virus, rescued by reverse genetics and inactivated by formalin, was shown to induce a dose-dependent protection correlating with neutralizing antibodies titers in Balb/c mice [86].

#### 4.2.5. Recombinant Viral Vector Vaccines

A recombinant canarypox viral vector expressing WNV PrM/E is approved for veterinary usage, requiring a two-dose regimen and an annual booster dose [39]. A live-attenuated recombinant equine herpes virus type 1 expressing WNV PrM/E was also proposed, but failed to induce strong immunity in horses [87]. A vesicular stomatitis virus (VSV) vector expressing WNV E glycoprotein has also been generated with either the Indiana (prime) or Chandipura (boost) surface G glycoprotein. This vector elicited strong humoral and cellular immune responses and protected mice from lethal challenge with the virulent Louisiana WNV strain LSU-AR01 [88]. A lentiviral HIV1-TRIP vector expressing the soluble form of the E glycoprotein from the ISR98 WNV strain elicited a long-lasting, protective and sterilizing humoral immunity, as early as one week after priming [89]. For safety reasons, a similar, but non-integrative, lentiviral vector was further generated that induced a robust B-cell response that fully protected mice from challenge with a lethal dose of WNV [90]. 

#### 4.2.6. Chimeric Vaccines

Two of the WNV vaccine candidates tested in clinical trials and described above are chimeric viruses (ChimeriVax-WN02 and Chimeric WN/DEN4). Another strategy used the attenuated DEN2 vaccine candidate (DEN2 PDK-53) as a backbone to generate chimeric DEN-WNV vaccines expressing the PrM/E of WNV NY99. Optimized constructs, including mutations that enhance cell-culture virus production, were shown to be highly attenuated and able to induce a protective immune response in mice [91].

#### 4.2.7. Live-Attenuated Vaccines Derived from Infectious Clones

A live attenuated WNV lineage 2 virus rescued from an infectious clone (WN1415) that contains a set of non-conservative mutations mostly in non-structural proteins genes has been shown to be highly attenuated and to induce a vigorous immune response at low doses that protected mice from the virulent NY99 strain [92]. Other live attenuated vaccines based on mutations of glycosylation sites on the E and NS1 proteins [93] or on selected mutations in the E protein known for attenuating other flaviviruses, such as the Japanese encephalitis virus (JEV) SA-14142 strain and the ChimeriVax-WN02 vaccines, combined to mutations in the 3’-non-coding stem-loop structure, have also been proposed [94]. Single amino-acid substitutions in the central portion of NS4B [95] and NS2A [96] proteins have been described to confer a highly attenuated phenotype in mice and could be introduced in WNV vaccine candidates.

**Table 3:** West Nile virus vaccines in pre-clinical development. EDIII, ectodomain III; VLP, virus-like particles; KUN, Kunjin; SRIP, single round infectious particles.

Vaccine	Strategy	Animal model
Truncated E (*Drosophila*)	-	Mice and horses [55,56]
Truncated E (Baculovirus)	-	Mice and Hamsters [52,53]
Truncated E -Nanoparticles LPS covered	Inflammasome-activating nanoparticle + LPS	Mice [60]
Truncated E –Nanoparticles	Inflammasome-activating nanoparticle + TLR9 Ligand	Mice [60]
CpG		
EDIII - bacterial flagellin	TLR5 Ligand	Mice [20]
Continuous B-cell epitope from EDIII + HSP60 p458 peptide as carrier	-	Mice [62]
VLP (bacteriophage AP205)-EDIII	-	Mice [63]
WNV VLP (insect cells)	-	Mice [64]
Retroviral VLP (gag)-EDIII	VLPs expressing EDIII	Mice [65]
Plasmid DNA-PrM/E	-	Mice and Horses [61]
Plasmid DNA PrM/E+ Inactivated vaccine	Synergetic effect	Mice [67]
Plasmid DNA EDIII + IL15 adjuvant plasmid	Enhance T-cell memory	Mice [68]
Plasmid DNA PrM/E + LAMP	Target MHC-II compartment	Mice [69]
WNV DNA lacking the capsid protein	Disable the formation of infectious particles	Mice [65]
Kunjin DNA vaccine infectious clone (RepliVax)	Non-replicating single-cycle vaccine	Mice, Hamsters and Monkeys [77,78]
Capsid-deleted Kunjin DNA vaccine + co-expression of C protein (SRIP)	*In vivo* expression of single round infectious particles	Mice and Horses [72]
Adenovirus vector expressing WNV C, PrM, E and NS1	Replication-incompetent vaccine	Mice [79]
Formalin-inactivated WNV vaccine (WN-VAX)	-	Mice [80]
Formaldehyde-inactivated vaccine Israel 98 (ISR98) strain	-	Geese [81]
H_2_O_2_-inactivated WN-KUN strain	Kunjin is naturally attenuated	Mice [80]
6 contiguous cDNA fragments encoding NY99 genome	Bipartite infectious clone	Mice [86]
VSV vector expressing WNV E	Live attenuated vaccine	Mice [88]
Lentiviral HIV1-vector sE ISR98 WNV	-	Mice [89]
Non-integrative lentiviral HIV1 vector sE ISR98 WNV	Increased safety compared to HIV1-TRIP	Mice [85]
Chimeric DEN2 backbone expressing WNV PrM/E	LAV, highly attenuated	Mice [90,91]
Infectious clone (WN1415)	LAV, highly attenuated	Mice [92]
Live attenuated virus (LAV) mutations in the E and NS1 glycosylation sites	LAV, highly attenuated	Mice [93]
Recombinant measles vaccine expressing sE ISR98 WNV	LAV, highly attenuated	Mice[100], Monkeys [102]

## 5. A WNV Vaccine Candidate Based on Recombinant Measles Vaccine

MV vaccine is a live-attenuated negative-stranded RNA virus. Produced on a large scale in many countries and easily distributed at low cost, this vaccine induces life-long immunity after a single injection. However, in spite of this excellent vaccine, measles appears very difficult to eliminate and still causes 160,000 deaths annually worldwide, mostly in poorly developed countries, but even Europe recently experienced severe measles outbreaks. Improving and maintaining measles vaccination for decades is essential to contain this most contagious disease. In this context, the use of recombinant live measles vaccines designed to immunize the pediatric or adult populations seems desirable. We have previously derived a vector from the live-attenuated Schwarz strain of MV [97] that very stably expresses large amounts of heterologous genetic material [98] and induces strong and long-term specific humoral and cellular immune responses in mice and macaques, even in presence of pre-existing immunity to measles [99,100,101,102,103,104]. This vector was adapted to several applications, including HIV, dengue and other emerging arboviral diseases. Depending on the target population and the epidemic situation, a recombinant MV vaccine might either replace the existing MV vaccine in the MMR vaccine (measles, mumps, and rubella) formulation for pediatric use or would only be used to boost the MV immunity and simultaneously vaccinate against the heterologous disease during adulthood. In the case of dengue or HIV, the recombinant vaccine could replace the existing MV vaccine in the MMR formulation. In the case of chikungunya or WNV, the vaccine is intended for adults, so it would not impact the use of MMR and could be given after the vaccinated individuals have completed their MV immunization schedule. A measles-HIV vaccine candidate currently under clinical development [105,106] has been evaluated in phase I clinical trial, demonstrating the preclinical and clinical safety and immunogenicity of this vector.

### 5.1. MVSchw-sE_WNV_ Vaccine

**Figure 1:**
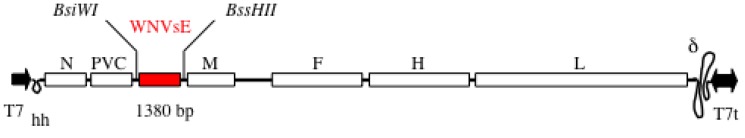
Schwarz/Moraten vaccine strain (MVSchw)-sE_WNV_ vector. The MV genes are indicated: N (nucleoprotein), PVC (phosphoprotein and V/C proteins), M (matrix), F (fusion), H (hemagglutinin), L (polymerase). T7 = T7 RNA polymerase promoter; hh = hammerhead ribozyme; T7t = T7 RNA polymerase terminator; ∂ = hepatitis delta virus (HDV) ribozyme; in red, the additional transcription units (ATU1) with the WNV sequence inserted. Total nucleotides in measles virus (MV) genome: 15,894.

The infectious cDNA of the widely used Schwarz/Moraten vaccine strain (MVSchw) allows the production of the Schwarz/Moraten vaccine without having to depend on the availability of seed stocks [97]. Additional transcription units (ATU) were introduced in the viral genome to turn it into a vector expressing foreign proteins (Figure 1). The expression of additional open-reading frames (ORFs) inserted in an ATU is controlled by cis-acting elements modeled after those present in the nucleocapsid /phosphoprotein boundary region (allowing for the necessary transient transcription stop upstream of the transgene, autonomous transcription, capping and polyadenylation of the transgene). Three vectors allow insertion of foreign genes in three different positions of the MV genome: one upstream the N gene, one between the P and M genes, and one between the H and L genes. Single vectors can be produced that express single antigens, and recombination of these vectors generates multiple vectors that express two or three different foreign genes. 

To generate MVSchw-sE_WNV_ recombinant vaccine, a cDNA encoding the truncated E glycoprotein lacking the transmembrane domain (sE) from the virulent IS98-ST1 strain of WNV was introduced into the ATU1 of the MV vector (Figure 1). The recombinant virus was produced by reverse genetics. After amplification in Vero cells, the progeny virus expressed large amounts of WNV E protein that was secreted from infected cells, and expression remained stable after 10 passages.

### 5.2. Immunogenicity in Mice

Genetically modified mice expressing the human CD46 MV receptor and lacking the IFN-α/β receptor (IFNAR) are needed to evaluate MV immunogenicity. We previously demonstrated that the molecularly cloned Schwarz MV induced strong immune responses in CD46/IFNAR mice that correlated to immune responses elicited in non-human primates [97]. We also showed the immunogenicity and protective efficacy of the recombinant MV vector in non-human primates [102,105,106], demonstrating the predictivity of CD46/IFNAR mice. Besides, IFNAR mice are highly susceptible to encephalitic flaviviruses, and we have shown that they survive only three days after intraperitoneal injection of 100 focus forming units of IS98-ST1 WNV virus [107]. We thus evaluated the efficacy of MVSchw-sE_WNV_ to protect from WNV encephalitis in this model. Immunization with a low dose protected mice from WNV challenge as early as eight days post-immunization. Six months after immunization, mice had still high levels of protective neutralizing antibodies, and the protection was maintained life-long after a single immunization. Protection was conferred by neutralizing antibodies as demonstrated by the passive transfer of only 2 µL of immune sera that protected naive mice from doses ranging from 100 to 100,000 focus-forming units of IS98-ST1 virus [100]. 

### 5.3. Immunogenicity in Non-Human Primates

#### 5.3.1. Squirrel Monkey: A New Primate Model for West Nile Virus Infection

A serosurvey performed in 2002 following an epidemic of WNV meningoencephalitis in southern Louisiana indicated that 36% of 1,692 captive rhesus monkeys (*Macaca mulatta*), pigtail macaques (*M. nemestrina*) and baboons (*Papio spp.*) from an outdoor breeding colony in the Tulane National Primate Center had WNV antibodies [108]. Comparison of these samples with previous sera collected before the epidemic indicated that the animals were naturally infected with WNV during the 2002 transmission season, although none of the infected animals developed clinical signs. This shows that non-human primates (NHP) can be naturally infected by WNV and may serve as a virus reservoir. Several non-human primates are susceptible to WNV infection, including rhesus macaques and baboons [109,110,111,112,113]. Intracranial or intranasal infection of rhesus and cynomolgus (*Macaca fasciacularis*) monkeys may result in encephalitis or death [114,115], whereas intravenous or subcutaneous infection is asymptomatic [109,110,111,112,113]. (please provide a clearer fig.)

**Figure 2:**
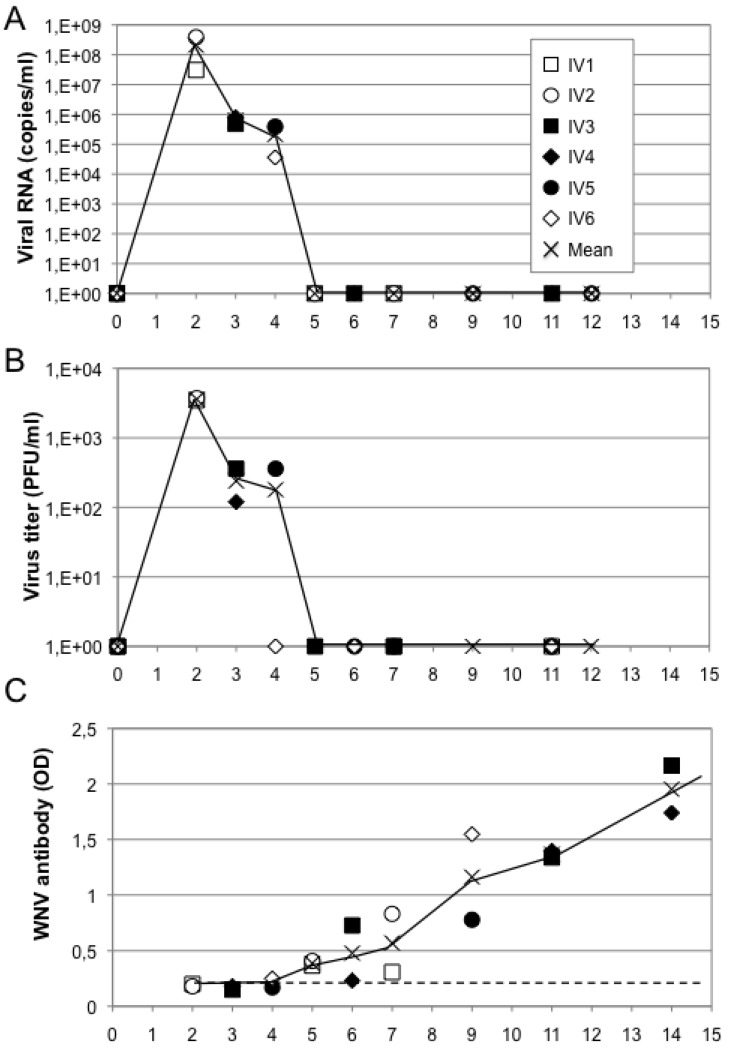
West Nile virus (WNV) replication in *Saimiri sciureus* squirrel monkeys. Six animals were inoculated intravenously with WNV (IV1 to IV6), and serum was collected to assess infection. (**A**) The presence of viral RNA was determined by quantitative real-time polymerase chain reaction. (**B**) Infectious virus was determined by the plaque formation assay. (**C**) WNV antibody levels were measured by enzyme-linked immunosorbent assay. Blood samples were collected on days 0, 2 and 5 for animals IV1 and IV2; on days 0, 3, 6 and 11 for animals IV3 and IV4; and on days 0, 4, 9 and 12 for animals IV5 and IV6. Monkeys were euthanized on day 7 for IV1 and IV2; on day 14 for IV3 and IV4; and on day 15 for IV5 and IV6. The black line represents the mean value for each time point. The dashed line in panel C represents the baseline determined from naive animal sera. Abbreviations: OD, optical density; pfu, plaque-forming units.

To be in compliance with the specific French recommendation for manipulating WNV *in vivo* in highly confined facilities, we developed a new NHP model based on a small animal that can be housed in small cages confined in biosafety level 4 (BSL4) facilities. We choose the New World squirrel monkey (*Saimiri sciureus*), weighing less than a kilo. After intravenous injection of 10^5^ pfu of IS98-ST1 WNV, a peak viremia was detected by qRT-PCR and infectivity plaque assay in all squirrel monkeys from day 2 to day 5 post-infection (Figure 2A,B), corresponding to a slight increase in body temperature [102]. 

No clinical symptoms or changes in body weight were observed. Viremia levels and duration were similar to those observed in other NHP models of WNV infection [109,110,111,112,113]. At sacrifice, viral RNA was found in the spleen, lymph nodes, liver, kidney and lungs of infected animals. The brain, spinal cord and meninges remained negative, indicating no productive infection in the central nervous system, as previously reported for other NHP intravenously infected. Histopathological analysis indicated some foci of infection in the liver, immune cell infiltration and endothelial inflammation. Half of the animals shed WNV in the urine. Serology analysis indicated a very rapid response to WNV, with specific antibodies detectable as early as five days post-inoculation and neutralizing antibodies by day 7 (Figure 2C). Squirrel monkeys can therefore be used as a new NHP model for WNV infection and assessment of vaccines candidates. 

#### 5.3.2. MV-WNV Induces Protective Immunity in Squirrel Monkeys

**Figure 3:**
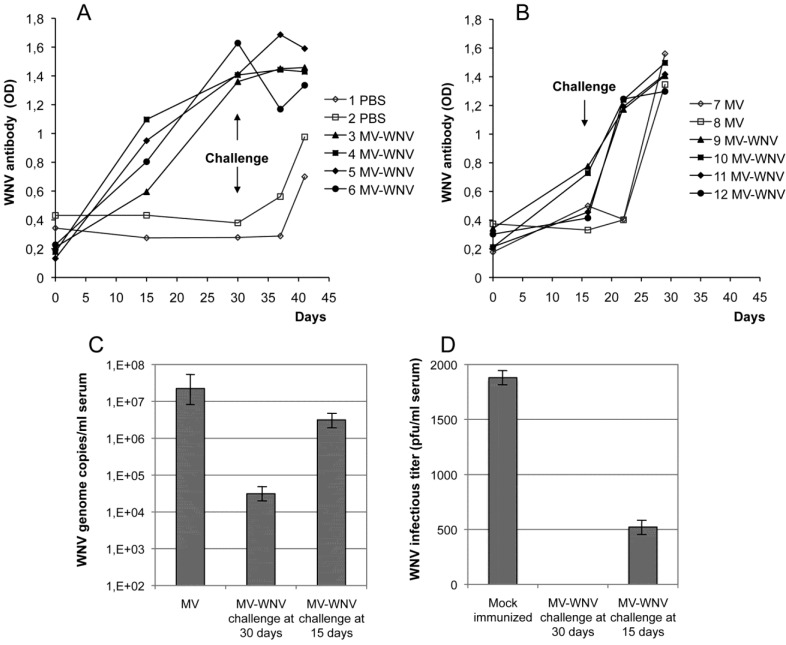
West Nile virus (WNV) challenge of squirrel monkeys immunized with MVSchw-sE_WNV_. Twelve monkeys immunized with MVSchw-sE_WNV_ and four monkeys mock immunized (with empty MV vaccine) or phosphate-buffered saline (PBS)) were challenged with WNV IS-98-ST1, either 15 or 30 days after immunization. (**A**) and (**B**): WNV-specific antibody levels were determined by enzyme-linked immunosorbent assay on the day of immunization, 15 days after immunization, the day of challenge, seven days after challenge and on the day of euthanasia. (**C**) The presence of WNV genomic RNA was determined by quantitative real-time polymerase chain reaction on day 2 after challenge in the serum of challenged animals. Animals 3, 4, 5 and 6 received one vaccination on day 30 before challenge; animals 9, 10, 11 and 12 received one vaccination on day 15 before challenge; and animals 1 and 2 (PBS) and 7 and 8 (MV) were mock infected. (**D**) The mean WNV titer in the serum of vaccinated animals, determined by the plaque formation assay at day 2 after challenge. Abbreviations: OD, optical density; pfu, plaque-forming units.

We used this model to evaluate the immunogenicity and protective efficacy of MVSchw-sE_WNV_ vaccine candidate. Squirrel monkeys were immunized intramuscularly with a single dose of 3.0 × 10^6^ tissue culture infective dose (TCID50) of vaccine and then challenged intravenously 15 or 30 days later with 10^5^ pfu of WNV IS98-ST1. No vaccine-associated adverse effects were observed, and as early as 15 days after immunization, WNV-specific IgG were detectable in six of eight animals. Additionally, ELISA titers increased sharply by day 30 post-immunization (Figure 3A,B). Neutralizing antibodies were also detected at day 15 and 30. At challenge, a slight increase in body temperature was recorded in control animals that received either or phosphate-buffered saline (PBS) or empty MV virus, but not in MVSchw-sE_WNV_ vaccinated animals. IgG antibodies titers in animals challenged 30 days post-immunization remained high and unchanged, while they were boosted in animals challenged 15 days post-immunization. In all vaccinated animals, the post-challenge neutralizing titers was one log higher than in control animals, indicating a boost of vaccine memory during WNV infection. A strong reduction of viremia was observed in both groups challenged 30 or 15 days after immunization (Figure 3C,D). In animals challenged 30 days after immunization, a three log reduction (99.8%) of WNV RNA copy number in plasma was observed, with no infectious titer detectable, whereas in the animals challenged only 15 days after immunization, a significant 10-fold reduction (85%) was still observed. Due to BSL4 constraints, long-term studies were not performed, but our previous studies demonstrate that the recombinant MV vector induces a long-term immune response. 

A combined MV-WNV vaccine might be administered to the pediatric naive population, but also to adolescent and adult populations with pre-existing MV immunity acquired after previous vaccination, which might prevent or reduce the efficacy of recombinant MV vaccine. However, we previously demonstrated that mice or macaques immunized with recombinant MV in the presence of MV pre-immunity are still efficiently immunized [99,103]. Yet, this point needs to be further evaluated in clinical trials. (please provide a clearer fig.)

### 5.4. Other Recombinant Measles-Arbovirus Vaccines

Despite decades of effort, no vaccine against dengue is available, although dengue disease is an increasing global health problem, threatening a third of the world’s population. This vaccine needs to confer protection against the four serotypes of dengue virus (DV), to provide long-lasting immunity, to be pediatric and produced at low cost on a large scale for global immunization. The strategies tested so far in humans are based on mixtures of four live attenuated viruses or four chimeric viruses (ChimeriVax^®^). These strategies are facing interference and formulation issues, leading to an unbalanced immune response against the four DV serotypes. To avoid such problems while still benefiting from the advantages of live vaccines, we designed a single MV vector expressing a tetravalent dengue antigen composed of the four DV serotypes. A tetravalent construct composed of the four EDIII of each of the four DV serotypes fused to the ectodomain of the membrane protein was inserted into the MV vector, and this vaccine candidate was proven immunogenic in mice [101,104]. Recently, an improved version of this vaccine was tested in cynomolgus macaques. Immunization with two doses of recombinant virus elicited neutralizing antibodies against the four DV serotypes in all vaccinated animals. After challenge with DV1 or DV4, a ten-fold boost in neutralizing titers was observed, associated with a strong reduction of DV viral load in the sera of vaccinated animals. The further development of this promising vaccine candidate is pursued now with an Austrian company (Themis). A combined measles-dengue vaccine might provide a one-shot approach to immunize children against both diseases where they co-exist.

Similarly, we recently described a recombinant MV vaccine expressing chikungunya virus (CHIKV) proteins [103]. CHIKV is a mosquito-transmitted alphavirus that recently reemerged in the Indian Ocean, India and Southeast-Asia, causing millions of cases of severe polyarthralgia. We generated a MV vector expressing CHIKV structural proteins, i.e., capsid, envelope E1 and E2 glycoproteins and two small peptides, E3 and 6K, allowing the formation of self-assembling virus-like particles (VLP) that mimic the alphavirus external structure. A single immunization with this vaccine induced high levels of neutralizing antibodies and protected CD46/IFNAR mice from lethal challenge with CHIKV homologous or heterologous circulating strains [103]. Supporting previous studies, passive transfer of immune sera conferred protection. Remarkably, pre-existing immunity to MV did not affect vaccine efficacy in this model. This vaccine was recently proven immunogenic in non-human primates and will be further developed into phase I study. This program revealed the capacity of MV vector to generate VLPs from a positive-stranded RNA virus. 

## 6. Concluding Remarks

After WNV introduction in the US in 1999 and an unprecedented peak epidemic in 2003, the number of cases dropped between 2008 and 2011. It was thus believed at that time that the virus would remain present at low-level transmission. However, in 2012, the US faced a new large WNV epidemic with a high rate of neurologic disease, indicating that the country may expect periodic WNV outbreaks in the next decades associated with human morbidity and mortality. Besides, the recent cases reported in Europe indicate that WNV appears to be expanding its geographical range. The development of an effective WNV vaccine is therefore more than ever urgent to stop WNV spread to new areas and to protect the populations at risk from neurologic complications. A number of approaches are currently being tested that seem promising. We believe that further large clinical development of WNV vaccine candidates must be encouraged and supported by big pharmaceutical companies. In this context, recombinant MV presents a number of advantages, such as the rapidity of immune response induction after a single immunization, long-term memory, safety and the possibility to produce large amounts of vaccine at low cost. 

## References

[1] Smithburn K.C.H., Burke A.W., Paul J.H. (1940). A neurotropic virus isolated from the blood of a native of Uganda. Am. J. Trop. Med. Hyg..

[2] Zeller H.G., Schuffenecker I. (2004). West Nile virus: an overview of its spread in Europe and the Mediterranean basin in contrast to its spread in the Americas. European journal of clinical microbiology & infectious diseases : official publication of the European Society of Clinical Microbiology.

[3] Malkinson M., Banet C., Weisman Y., Pokamunski S., King R., Drouet M.T., Deubel V. (2002). Introduction of West Nile virus in the Middle East by migrating white storks. Emerg. Infect. Dis..

[4] Murray K.O., Mertens E., Despres P. (2010). West Nile virus and its emergence in the United States of America. Veterinary research.

[5] Prevention, Centers for Disease Control and Prevention (CDC) West Nile Virus (WNV) Human Infections Reported to ArboNET, by State, United States, 2012 (as of December 11, 2012). 2012 [cited 2012 21–02–2013]. http://www.cdc.gov/ncidod/dvbid/westnile/surv&controlCaseCount12_detailed.htm.

[6] Prevention, European Center for Disease Control and Prevention (ECDC). http://ecdc.europa.eu/en/healthtopics/west_nile_fever/West-Nile-fever-maps/Pages/index.aspx.

[7] Allain J.P., Stramer S.L., Carneiro-Proietti A.B., Martins M.L., Lopes da Silva S.N., Ribeiro M., Proietti F.A., Reesink H.W. (2009). Transfusion-transmitted infectious diseases. Biologicals: J. Int. Ass. of Biol. Stand..

[8] Hinckley A.F., O'Leary D.R., Hayes E.B. (2007). Transmission of West Nile virus through human breast milk seems to be rare. Pediatrics.

[9] (2004). Interim guidelines for the evaluation of infants born to mothers infected with West Nile virus during pregnancy. MMWR.

[10] Mackenzie J.S., Williams D.T. (2009). The zoonotic flaviviruses of southern, south-eastern and eastern Asia, and Australasia: the potential for emergent viruses. Zoon. Publ. Health.

[11] Lanciotti R.S., Roehrig J.T., Deubel V., Smith J., Parker M., Steele K., Crise B., Volpe K.E., Crabtree M.B., Scherret J.H., Hall R.A., MacKenzie J.S., Cropp C.B., Panigrahy B., Ostlund E., Schmitt B., Malkinson M., Banet C., Weissman J., Komar N., Savage H.M., Stone W., McNamara T., Gubler D.J. (1999). Origin of the West Nile virus responsible for an outbreak of encephalitis in the northeastern United States. Science.

[12] Lanciotti R.S., Ebel G.D., Deubel V., Kerst A.J., Murri S., Meyer R., Bowen M., McKinney N., Morrill W.E., Crabtree M.B., Kramer L.D., Roehrig J.T. (2002). Complete genome sequences and phylogenetic analysis of West Nile virus strains isolated from the United States, Europe, and the Middle East. Virology.

[13] Papa A. (2012). West Nile virus infections in Greece: an update. Expert Rev. of Anti-Infect. Ther..

[14] Papa A., Politis C., Tsoukala A., Eglezou A., Bakaloudi V., Hatzitaki M., Tsergouli K. (2012). West Nile virus lineage 2 from blood donor, Greece. Emerg. Infect. Dis..

[15] Bakonyi T., Hubalek Z., Rudolf I., Nowotny N. (2005). Novel flavivirus or new lineage of West Nile virus, central Europe. Emerg. Infect. Dis..

[16] Bondre V.P., Jadi R.S., Mishra A.C., Yergolkar P.N., Arankalle V.A. (2007). West Nile virus isolates from India: evidence for a distinct genetic lineage. J. Gen. Virol..

[17] Arnold C. (2012). West Nile virus bites back. Lancet Neurol..

[18] Beasley D.W. (2011). Vaccines and immunotherapeutics for the prevention and treatment of infections with West Nile virus. Immunotherapy.

[19] Martina B.E., Koraka P., van den Doel P., van Amerongen G., Rimmelzwaan G.F., Osterhaus A.D. (2008). Immunization with West Nile virus envelope domain III protects mice against lethal infection with homologous and heterologous virus. Vaccine.

[20] McDonald W.F., Huleatt J.W., Foellmer H.G., Hewitt D., Tang J., Desai P., Price A., Jacobs A., Takahashi V.N., Huang Y., Nakaar V., Alexopoulou L., Fikrig E., Powell T.J. (2007). A West Nile virus recombinant protein vaccine that coactivates innate and adaptive immunity. J. Infect. Dis..

[21] Heinz F.X., Stiasny K. (2012). Flaviviruses and flavivirus vaccines. Vaccine.

[22] Nybakken G.E., Nelson C.A., Chen B.R., Diamond M.S., Fremont D.H. (2006). Crystal structure of the West Nile virus envelope glycoprotein. J. Virol..

[23] Kanai R., Kar K., Anthony K., Gould L.H., Ledizet M., Fikrig E., Marasco W.A., Koski R.A., Modis Y. (2006). Crystal structure of west nile virus envelope glycoprotein reveals viral surface epitopes. J. Virol..

[24] Modis Y., Ogata S., Clements D., Harrison S.C. (2003). A ligand-binding pocket in the dengue virus envelope glycoprotein. Proc. Natl. Acad. Sci. U.S.A..

[25] Modis Y., Ogata S., Clements D., Harrison S.C. (2004). Structure of the dengue virus envelope protein after membrane fusion. Nature.

[26] Nayak V., Dessau M., Kucera K., Anthony K., Ledizet M., Modis Y. (2009). Crystal structure of dengue virus type 1 envelope protein in the postfusion conformation and its implications for membrane fusion. J. Virol..

[27] Rey F.A., Heinz F.X., Mandl C., Kunz C., Harrison SC. (1995). The envelope glycoprotein from tick-borne encephalitis virus at 2 A resolution. Nature.

[28] Throsby M., Geuijen C., Goudsmit J., Bakker A.Q., Korimbocus J., Kramer R.A., Clijsters-van der Horst M., de Jong M., Jongeneelen M., Thijsse S., Smit R., Visser T.J., Bijl N., Marissen W.E., Loeb M., Kelvin D.J., Preiser W., ter Meulen J., de Kruif J. (2006). Isolation and characterization of human monoclonal antibodies from individuals infected with West Nile Virus. J. Virol..

[29] Thomas S., Redfern J.B., Lidbury B.A., Mahalingam S. (2006). Antibody-dependent enhancement and vaccine development. Expert. Rev. Vaccines.

[30] Diamond M.S., Pierson T.C., Fremont D.H. (2008). The structural immunology of antibody protection against West Nile virus. Immunol. Rev..

[31] Pierson T.C., Fremont D.H., Kuhn R.J., Diamond M.S. (2008). Structural insights into the mechanisms of antibody-mediated neutralization of flavivirus infection: implications for vaccine development. Cell host & microbe.

[32] White L.J., Sariol C.A., Mattocks M.D., Wahala M.P.B.W., Yingsiwaphat V., Collier M.L., Whitley J., Mikkelsen R., Rodriguez I.V., Martinez M.I., de Silva A., Johnston R.E. (2013). An alphavirus vector-based tetravalent dengue vaccine induces a rapid and protective immune response in macaques that differs qualitatively from immunity induced by live virus infection. J. Virol..

[33] de Alwis R., Smith S.A., Olivarez N.P., Messer W.B., Huynh J.P., Wahala W.M., White L.J., Diamond M.S., Baric R.S., Crowe J.E., de Silva A.M. (2012). Identification of human neutralizing antibodies that bind to complex epitopes on dengue virions. Proc. Natl. Acad. Sci. U.S.A..

[34] Kaufmann B., Vogt M.R., Goudsmit J., Holdaway H.A., Aksyuk A.A., Chipman P.R., Kuhn R.J., Diamond M.S., Rossmann M.G. (2010). Neutralization of West Nile virus by cross-linking of its surface proteins with Fab fragments of the human monoclonal antibody CR4354. Proc. Natl. Acad. Sci. U.S.A..

[35] Vogt M.R., Moesker B., Goudsmit J., Jongeneelen M., Austin S.K., Oliphant T., Nelson S., Pierson T.C., Wilschut J., Throsby M., Diamond M.S. (2009). Human monoclonal antibodies against West Nile virus induced by natural infection neutralize at a postattachment step. J. Virol..

[36] Lok S.M., Kostyuchenko V., Nybakken G.E., Holdaway H.A., Battisti A.J., Sukupolvi-Petty S., Sedlak D., Fremont D.H., Chipman P.R., Roehrig J.T., Diamond M.S., Kuhn R.J., Rossmann M.G. (2008). Binding of a neutralizing antibody to dengue virus alters the arrangement of surface glycoproteins. Nat. Struct. Mol. Biol..

[37] Ng T., Hathaway D., Jennings N., Champ D., Chiang Y.W., Chu H.J. (2003). Equine vaccine for West Nile virus. Dev. Biol..

[38] El Garch H., Minke J.M., Rehder J., Richard S., Edlund Toulemonde C., Dinic S., Andreoni C., Audonnet J.C., Nordgren R., Juillard V. (2008). A West Nile virus (WNV) recombinant canarypox virus vaccine elicits WNV-specific neutralizing antibodies and cell-mediated immune responses in the horse. Vet. Immunol. Immunopathol..

[39] Karaca K., Bowen R., Austgen L.E., Teehee M., Siger L., Grosenbaugh D., Loosemore L., Audonnet J.C., Nordgren R., Minke J.M. (2005). Recombinant canarypox vectored West Nile virus (WNV) vaccine protects dogs and cats against a mosquito WNV challenge. Vaccine.

[40] Monath T.P., Liu J., Kanesa-Thasan N., Myers G.A., Nichols R., Deary A., McCarthy K., Johnson C., Ermak T., Shin S., Arroyo J., Guirakhoo F., Kennedy J.S., Ennis F.A., Green S., Bedford P. (2006). A live, attenuated recombinant West Nile virus vaccine. Proc. Natl. Acad. Sci. U.S.A..

[41] Smith H. L., Monath T.P., Pazoles P., Rothman A.L., Casey D.M., Terajima M., Ennis F.A., Guirakhoo F., Green S. (2011). Development of antigen-specific memory CD8+ T cells following live-attenuated chimeric West Nile virus vaccination. J. Infect. Dis..

[42] Guy B., Guirakhoo F., Barban V., Higgs S., Monath T.P., Lang J. (2010). Preclinical and clinical development of YFV 17D-based chimeric vaccines against dengue, West Nile and Japanese encephalitis viruses. Vaccine.

[43] Dayan G.H., Bevilacqua J., Coleman D., Buldo A., Risi G. (2012). Phase II, dose ranging study of the safety and immunogenicity of single dose West Nile vaccine in healthy adults >/= 50 years of age. Vaccine.

[44] Pletnev A.G., Swayne D.E., Speicher J., Rumyantsev A.A., Murphy B.R. (2006). Chimeric West Nile/dengue virus vaccine candidate: preclinical evaluation in mice, geese and monkeys for safety and immunogenicity. Vaccine.

[45] De Filette M., Ulbert S., Diamond M., Sanders N.N. (2012). Recent progress in West Nile virus diagnosis and vaccination. Vet. Res..

[46] Hanley K.A., Goddard L.B., Gilmore L.E., Scott T.W., Speicher J., Murphy B.R., Pletnev A.G. (2005). Infectivity of West Nile/dengue chimeric viruses for West Nile and dengue mosquito vectors. Vector Borne Zoonotic. Dis..

[47] Ledgerwood J.E., Pierson T.C., Hubka S.A., Desai N., Rucker S., Gordon I.J., Enama M.E., Nelson S., Nason M., Gu W., Bundrant N., Koup R.A., Bailer R.T., Mascola J.R., Nabel G.J., Graham B.S. (2011). A West Nile virus DNA vaccine utilizing a modified promoter induces neutralizing antibody in younger and older healthy adults in a phase I clinical trial. J. Infect. Dis..

[48] Martin J.E., Pierson T.C., Hubka S., Rucker S., Gordon I.J., Enama M.E., Andrews C.A., Xu Q., Davis B.S., Nason M., Fay M., Koup R.A., Roederer M., Bailer R.T., Gomez P. L., Mascola J.R., Chang G.J., Nabel G.J., Graham B.S. (2007). A West Nile virus DNA vaccine induces neutralizing antibody in healthy adults during a phase 1 clinical trial. J. Infect. Dis..

[49] Lieberman M.M., Clements D.E., Ogata S., Wang G., Corpuz G., Wong T., Martyak T., Gilson L., Coller B.A., Leung J., Watts D.M., Tesh R.B., Siirin M., Travassos da Rosa A., Humphreys T., Weeks-Levy C. (2007). Preparation and immunogenic properties of a recombinant West Nile subunit vaccine. Vaccine.

[50] Lieberman M.M., Nerurkar V.R., Luo H., Cropp B., Carrion R., de la Garza M., Coller B.A., Clements D., Ogata S., Wong T., Martyak T., Weeks-Levy C. (2009). Immunogenicity and protective efficacy of a recombinant subunit West Nile virus vaccine in rhesus monkeys. Clin. Vaccine Immunol..

[51] Watts D.M., Tesh R.B., Siirin M., Rosa A.T., Newman P.C., Clements D.E., Ogata S., Coller B.A., Weeks-Levy C., Lieberman M.M. (2007). Efficacy and durability of a recombinant subunit West Nile vaccine candidate in protecting hamsters from West Nile encephalitis. Vaccine.

[52] Jarvi S.I., Hu D., Misajon K., Coller B.A., Wong T., Lieberman M.M. (2013). Vaccination of captive nene (Branta sandvicensis) against West Nile virus using a protein-based vaccine (WN-80E). J. Wildlife Dis..

[B53-viruses-05-02384] 53.database, N. c. t. clinicaltrials.gov

[54] Coller B.-A., Weeks-Levy C., Ogata S. (2012). RECOMBINANT SUBUNIT WEST NILE VIRUS VACCINE FOR PROTECTION OF HUMAN SUBJECTS. Patent application number: 20120141520.

[55] Wang T., Anderson J.F., Magnarelli L.A., Wong S.J., Koski R.A., Fikrig E. (2001). Immunization of mice against West Nile virus with recombinant envelope protein. J. Immunol..

[56] Ledizet M., Kar K., Foellmer H.G., Wang T., Bushmich S.L., Anderson J.F., Fikrig E., Koski R.A. (2005). A recombinant envelope protein vaccine against West Nile virus. Vaccine.

[57] Bonafe N., Rininger J.A., Chubet R.G., Foellmer H.G., Fader S., Anderson J.F., Bushmich S.L., Anthony K., Ledizet M., Fikrig E., Koski R.A., Kaplan P. (2009). A recombinant West Nile virus envelope protein vaccine candidate produced in Spodoptera frugiperda expresSF+ cells. Vaccine.

[58] Zhu B., Ye J., Lu P., Jiang R., Yang X., Fu Z.F., Chen H., Cao S. (2012). Induction of antigen-specific immune responses in mice by recombinant baculovirus expressing premembrane and envelope proteins of West Nile virus. Virol. J..

[59] Demento S.L., Eisenbarth S.C., Foellmer H.G., Platt C., Caplan M.J., Mark Saltzman W., Mellman I., Ledizet M., Fikrig E., Flavell R.A., Fahmy T.M. (2009). Inflammasome-activating nanoparticles as modular systems for optimizing vaccine efficacy. Vaccine.

[60] Demento S.L., Bonafe N., Cui W., Kaech S.M., Caplan M.J., Fikrig E., Ledizet M., Fahmy T.M. (2010). TLR9-targeted biodegradable nanoparticles as immunization vectors protect against West Nile encephalitis. J. Immunol..

[61] Chu J.H., Chiang C.C., Ng M.L. (2007). Immunization of flavivirus West Nile recombinant envelope domain III protein induced specific immune response and protection against West Nile virus infection. J. Immunol..

[62] Gershoni-Yahalom O., Landes S., Kleiman-Shoval S., Ben-Nathan D., Kam M., Lachmi B.E., Khinich Y., Simanov M., Samina I., Eitan A., Cohen I.R., Rager-Zisman B., Porgador A. (2010). Chimeric vaccine composed of viral peptide and mammalian heat-shock protein 60 peptide protects against West Nile virus challenge. Immunology.

[63] Spohn G., Jennings G.T., Martina B.E., Keller I., Beck M., Pumpens P., Osterhaus A.D., Bachmann M.F. (2010). A VLP-based vaccine targeting domain III of the West Nile virus E protein protects from lethal infection in mice. Virol. J..

[64] Qiao M., Ashok M., Bernard K.A., Palacios G., Zhou Z.H., Lipkin W.I., Liang T.J. (2004). Induction of sterilizing immunity against West Nile Virus (WNV), by immunization with WNV-like particles produced in insect cells. J. Infect. Dis..

[65] Chua A.J., Vituret C., Tan M.L., Gonzalez G., Boulanger P., Ng M.L., Hong S.S. (2013). A novel platform for virus-like particle-display of flaviviral envelope domain III: induction of Dengue and West Nile virus neutralizing antibodies. Virol. J..

[66] Davis B.S., Chang G.J., Cropp B., Roehrig J.T., Martin D.A., Mitchell C.J., Bowen R., Bunning M.L. (2001). West Nile virus recombinant DNA vaccine protects mouse and horse from virus challenge and expresses in vitro a noninfectious recombinant antigen that can be used in enzyme-linked immunosorbent assays. J. Virol..

[67] Ishikawa T., Takasaki T., Kurane I., Nukuzuma S., Kondo T., Konishi E. (2007). Co-immunization with West Nile DNA and inactivated vaccines provides synergistic increases in their immunogenicities in mice. Microbes. Infect..

[68] Ramanathan M.P., Kutzler M.A., Kuo Y.C., Yan J., Liu H., Shah V., Bawa A., Selling B., Sardesai N.Y., Kim J.J., Weiner D.B. (2009). Coimmunization with an optimized IL15 plasmid adjuvant enhances humoral immunity via stimulating B cells induced by genetically engineered DNA vaccines expressing consensus JEV and WNV E DIII. Vaccine.

[69] Anwar A., Chandrasekaran A., Ng M.L., Marques E., August J.T. (2005). West Nile premembrane-envelope genetic vaccine encoded as a chimera containing the transmembrane and cytoplasmic domains of a lysosome-associated membrane protein: increased cellular concentration of the transgene product, targeting to the MHC II compartment, and enhanced neutralizing antibody response. Virology.

[70] Seregin A., Nistler R., Borisevich V., Yamshchikov G., Chaporgina E., Kwok C.W., Yamshchikov V. (2006). Immunogenicity of West Nile virus infectious DNA and its noninfectious derivatives. Virology.

[71] Hall R.A., Nisbet D.J., Pham K.B., Pyke A.T., Smith G.A., Khromykh A.A. (2003). DNA vaccine coding for the full-length infectious Kunjin virus RNA protects mice against the New York strain of West Nile virus. Proc. Natl. Acad. Sci. U.S.A..

[72] Chang D.C., Liu W.J., Anraku I., Clark D.C., Pollitt C.C., Suhrbier A., Hall R.A., Khromykh A.A. (2008). Single-round infectious particles enhance immunogenicity of a DNA vaccine against West Nile virus. Nat. Biotechnol..

[73] Widman D.G., Ishikawa T., Winkelmann E.R., Infante E., Bourne N., Mason P.W. (2009). RepliVAX WN, a single-cycle flavivirus vaccine to prevent West Nile disease, elicits durable protective immunity in hamsters. Vaccine.

[74] Widman D.G., Ishikawa T., Giavedoni L.D., Hodara V.L., Garza Mde L., Montalbo J.A., Travassos Da Rosa A.P., Tesh R.B., Patterson J.L., Carrion R., Bourne N., Mason P. W. (2010). Evaluation of RepliVAX WN, a single-cycle flavivirus vaccine, in a non-human primate model of West Nile virus infection. Am. J. Trop. Med. Hyg..

[75] Suzuki R., Fayzulin R., Frolov I., Mason P.W. (2008). Identification of mutated cyclization sequences that permit efficient replication of West Nile virus genomes: use in safer propagation of a novel vaccine candidate. J. Virol..

[76] Widman D.G., Frolov I., Mason P.W. (2008). Third-generation flavivirus vaccines based on single-cycle, encapsidation-defective viruses. Adv. Virus Res..

[77] Ishikawa T., Widman D.G., Bourne N., Konishi E., Mason P.W. (2008). Construction and evaluation of a chimeric pseudoinfectious virus vaccine to prevent Japanese encephalitis. Vaccine.

[78] Widman D.G., Ishikawa T., Fayzulin R., Bourne N., Mason P.W. (2008). Construction and characterization of a second-generation pseudoinfectious West Nile virus vaccine propagated using a new cultivation system. Vaccine.

[79] Schepp-Berglind J., Luo M., Wang D., Wicker J.A., Raja N.U., Hoel B.D., Holman D.H., Barrett A.D., Dong J.Y. (2007). Complex adenovirus-mediated expression of West Nile virus C, PreM, E, and NS1 proteins induces both humoral and cellular immune responses. Clin. Vaccine Immunol..

[80] Lim C.K., Takasaki T., Kotaki A., Kurane I. (2008). Vero cell-derived inactivated West Nile (WN) vaccine induces protective immunity against lethal WN virus infection in mice and shows a facilitated neutralizing antibody response in mice previously immunized with Japanese encephalitis vaccine. Virology.

[81] Samina I., Havenga M., Koudstaal W., Khinich Y., Koldijk M., Malkinson M., Simanov M., Perl S., Gijsbers L., Weverling G.J., Uytdehaag F., Goudsmit J. (2007). Safety and efficacy in geese of a PER.C6-based inactivated West Nile virus vaccine. Vaccine.

[82] Scherret J.H., Mackenzie J.S., Hall R.A., Deubel V., Gould E.A. (2002). Phylogeny and molecular epidemiology of West Nile and Kunjin viruses. Curr. Top. Microbiol. Immunol..

[83] Scherret J.H., Poidinger M., Mackenzie J.S., Broom A.K., Deubel V., Lipkin W.I., Briese T., Gould E.A., Hall R.A. (2001). The relationships between West Nile and Kunjin viruses. Emerg. Infect. Dis..

[84] Gray T.J., Burrow J.N., Markey P.G., Whelan P.I., Jackson J., Smith D.W., Currie B.J. (2011). West nile virus (Kunjin subtype) disease in the northern territory of Australia--a case of encephalitis and review of all reported cases. Am. J. Trop. Med. Hyg..

[85] Pinto A.K., Richner J.M., Poore E.A., Patil P.P., Amanna I.J., Slifka M.K., Diamond M.S. (2013). A Hydrogen Peroxide-Inactivated Virus Vaccine Elicits Humoral and Cellular Immunity and Protects against Lethal West Nile Virus Infection in Aged Mice. J. Virol..

[86] Orlinger K.K., Holzer G.W., Schwaiger J., Mayrhofer J., Schmid K., Kistner O., Noel Barrett P., Falkner F.G. (2010). An inactivated West Nile Virus vaccine derived from a chemically synthesized cDNA system. Vaccine.

[87] Rosas C.T., Tischer B.K., Perkins G.A., Wagner B., Goodman L.B., Osterrieder N. (2007). Live-attenuated recombinant equine herpesvirus type 1 (EHV-1) induces a neutralizing antibody response against West Nile virus (WNV). Virus Res..

[88] Iyer A.V., Pahar B., Boudreaux M.J., Wakamatsu N., Roy A.F., Chouljenko V.N., Baghian A., Apetrei C., Marx P.A., Kousoulas K.G. (2009). Recombinant vesicular stomatitis virus-based west Nile vaccine elicits strong humoral and cellular immune responses and protects mice against lethal challenge with the virulent west Nile virus strain LSU-AR01. Vaccine.

[89] Iglesias M.C., Frenkiel M.P., Mollier K., Souque P., Despres P., Charneau P. (2006). A single immunization with a minute dose of a lentiviral vector-based vaccine is highly effective at eliciting protective humoral immunity against West Nile virus. J. Gene Med..

[90] Coutant F., Frenkiel M.P., Despres P., Charneau P. (2008). Protective antiviral immunity conferred by a nonintegrative lentiviral vector-based vaccine. PLoS ONE.

[91] Huang C.Y., Silengo S.J., Whiteman M.C., Kinney R.M. (2005). Chimeric dengue 2 PDK-53/West Nile NY99 viruses retain the phenotypic attenuation markers of the candidate PDK-53 vaccine virus and protect mice against lethal challenge with West Nile virus. J. Virol..

[92] Yamshchikov G., Borisevich V., Seregin A., Chaporgina E., Mishina M., Mishin V., Kwok C.W., Yamshchikov V. (2004). An attenuated West Nile prototype virus is highly immunogenic and protects against the deadly NY99 strain: a candidate for live WN vaccine development. Virology.

[93] Whiteman M.C., Li L., Wicker J.A., Kinney R.M., Huang C., Beasley D.W., Chung K.M., Diamond M.S., Solomon T., Barrett A.D. (2010). Development and characterization of non-glycosylated E and NS1 mutant viruses as a potential candidate vaccine for West Nile virus. Vaccine.

[94] Yu L., Robert Putnak J., Pletnev A.G., Markoff L. (2008). Attenuated West Nile viruses bearing 3'SL and envelope gene substitution mutations. Vaccine.

[95] Wicker J.A., Whiteman M.C., Beasley D.W., Davis C.T., Zhang S., Schneider B.S., Higgs S., Kinney R.M., Barrett A.D. (2006). A single amino acid substitution in the central portion of the West Nile virus NS4B protein confers a highly attenuated phenotype in mice. Virology.

[96] Liu W.J., Wang X.J., Clark D.C., Lobigs M., Hall R.A., Khromykh A.A. (2006). A single amino acid substitution in the West Nile virus nonstructural protein NS2A disables its ability to inhibit alpha/beta interferon induction and attenuates virus virulence in mice. J. Virol..

[97] Combredet C., Labrousse V., Mollet L., Lorin C., Delebecque F., Hurtrel B., McClure H., Feinberg M.B., Brahic M., Tangy F. (2003). A molecularly cloned Schwarz strain of measles virus vaccine induces strong immune responses in macaques and transgenic mice. J. Virol..

[98] Tangy F., Lucas M., Navarro-Sanchez E., Frenkiel M., Combredet C., Despres P. Chimeric poly peptides and their therapeutic use against a flaviviridae infection.

[99] Lorin C., Mollet L., Delebecque F., Combredet C., Charneau P., Hurtrel B., Brahic M., Tangy F. (2004). A Single Injection of Recombinant Measles Vaccines Expressing HIV-1 Clade B Envelope Glycoproteins Induces Neutralizing Antibodies and Cellular Immune Responses to HIV. J. Virol..

[100] Desprès P., Combredet C., Frenkiel M., Lorin C., Brahic M., Tangy F. (2005). Live measles vaccine expressing the secreted form of the West Nile virus envelope glycoprotein protects against West Nile virus encephalitis. J. Infect. Dis..

[101] Brandler S., Lucas-Hourani M., Moris A., Frenkiel M.P., Combredet C., Fevrier M., Bedouelle H., Schwartz O., Despres P., Tangy F. (2007). Pediatric Measles Vaccine Expressing a Dengue Antigen Induces Durable Serotype-specific Neutralizing Antibodies to Dengue Virus. PLoS neglected tropical diseases.

[102] Brandler S., Marianneau P., Loth P., Lacote S., Combredet C., Frenkiel M.P., Despres P., Contamin H., Tangy F. (2012). Measles vaccine expressing the secreted form of west nile virus envelope glycoprotein induces protective immunity in squirrel monkeys, a new model of west nile virus infection. J. Infect. Dis..

[103] Brandler S., Ruffie C., Combredet C., Brault J.B., Najburg V., Prevost M.C., Habel A., Tauber E., Despres P., Tangy F. A recombinant measles vaccine expressing chikungunya virus-like particles is strongly immunogenic and protects mice from lethal challenge with chikungunya virus. Vaccine.

[104] Brandler S., Ruffie C., Najburg V., Frenkiel M.P., Bedouelle H., Despres P., Tangy F. (2010). Pediatric measles vaccine expressing a dengue tetravalent antigen elicits neutralizing antibodies against all four dengue viruses. Vaccine.

[105] Stebbings R., Fevrier M., Li B., Lorin C., Koutsoukos M., Mee E., Rose N., Hall J., Page M., Almond N., Voss G., Tangy F. (2012). Immunogenicity of a recombinant measles-HIV-1 clade B candidate vaccine. PLoS ONE.

[106] Lorin C., Segal L., Mols J., Morelle D., Bourguignon P., Rovira O., Mettens P., Silvano J., Dumey N., Le Goff F., Koutsoukos M., Voss G., Tangy F. (2012). Toxicology, biodistribution and shedding profile of a recombinant measles vaccine vector expressing HIV-1 antigens, in cynomolgus macaques. Naunyn-Schmiedeberg's Arch. Pharmacol..

[107] Lucas M., Mashimo T., Frenkiel M.-P., Simon-Chazottes D., Montagutelli X., Ceccaldi P.-E., Guénet J.-L., Desprès P. (2003). Infection of mouse neurons by West Nile virus is modulated by the interferon-inducible 2’-5’ oligoadenylate synthetase 1b protein. Immun. Cell Biol..

[108] Ratterree M.S., da Rosa A.P., Bohm R.P., Cogswell F.B., Phillippi K.M., Caillouet K., Schwanberger S., Shope R.E., Tesh R.B. (2003). West Nile virus infection in nonhuman primate breeding colony, concurrent with human epidemic, southern Louisiana. Emerg. Infect. Dis..

[109] Arroyo J., Miller C., Catalan J., Myers G.A., Ratterree M.S., Trent D.W., Monath T.P. (2004). ChimeriVax-West Nile virus live-attenuated vaccine: preclinical evaluation of safety, immunogenicity, and efficacy. J. Virol..

[110] Ratterree M., Gutierrez R., Travassos da Rosa A., Dille B., Beasley D., Bohm R., Desai S., Didier P., Bikenmeyer L., Dawson G., Leary T., Schochetman G., Phillippi-Falkenstein K., Arroyo J., Barrett A., Tesh R. (2004). Experimental infection of rhesus macaques with West Nile virus: level and duration of viremia and kinetics of the antibody response after infection. J. Infect. Dis..

[111] Wertheimer A.M., Uhrlaub J.L., Hirsch A., Medigeshi G., Sprague J., Legasse A., Wilk J., Wiley C.A., Didier P., Tesh R.B., Murray K.O., Axthelm M.K., Wong S.W., Nikolich-Zugich J. Immune response to the West Nile virus in aged non-human primates. PLoS ONE.

[112] Pogodina V., Frolova M., Malenko G., Fokina G., Koreshkova G., Kiseleva L., Bochkova N., Ralph N., Related Articles L. A. (1983). Study on West Nile virus persistence in monkeys. Arch. Virol..

[113] Wolf R.F., Papin J.F., Hines-Boykin R., Chavez-Suarez M., White G.L., Sakalian M., Dittmer D.P. (2006). Baboon model for West Nile virus infection and vaccine evaluation. Virology.

[114] Nathanson N., Davis M., Thind I.S., Price W.H. (1966). Histological studies of the monkey neurovirulence of group B arboviruses. II. Selection of indicator centers. Am. J. Epidemiol..

[115] Manuelidis E.E. (1956). Neuropathology of experimental West Nile virus infection in monkeys. J. Neuropath. Exp. Neur..

